# Supplementary material for: Changes in ALBI Score and PIVKA-II within Three Months after Commencing Atezolizumab Plus Bevacizumab Treatment Affect Overall Survival in Patients with Unresectable Hepatocellular Carcinoma
Source: Cancers (Basel). 2022 Dec 10;14(24):6089. doi: 10.3390/cancers14246089 (PMC9776967; doi:10.3390/cancers14246089)
Supplement: Supplementary file 1 [file cancers-14-06089-s001.zip › cancers-2086371-supplementary.pdf]

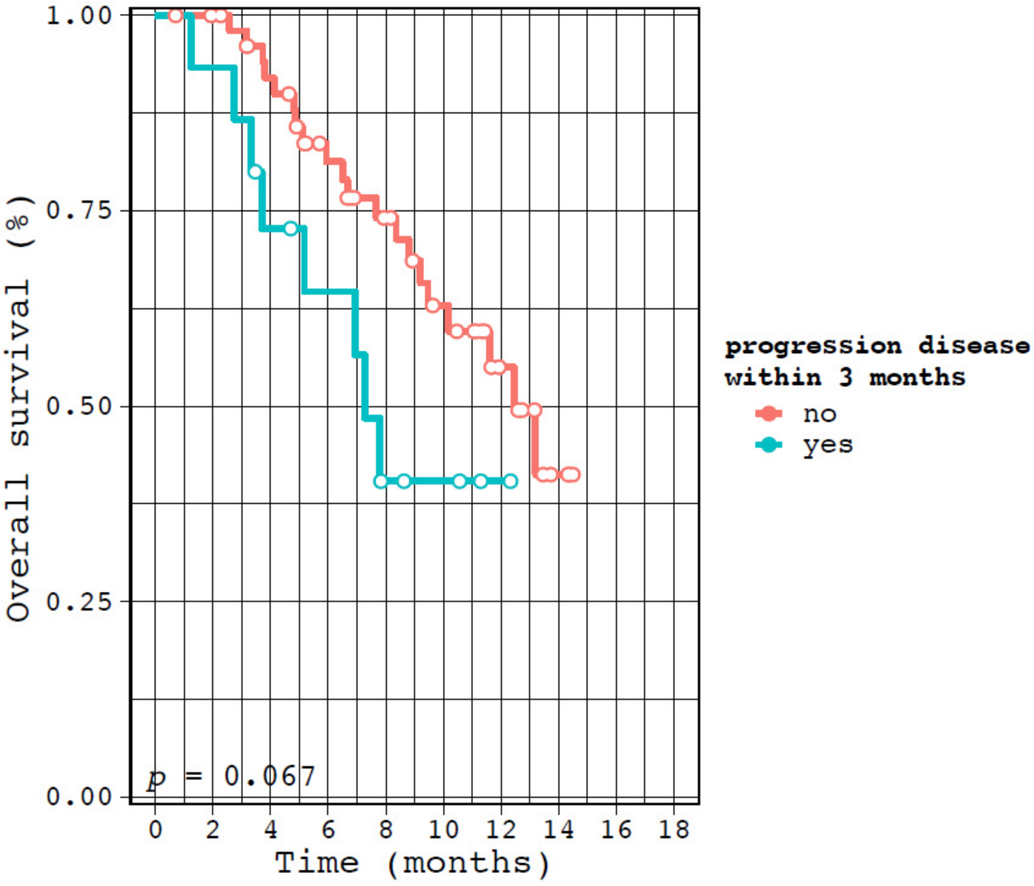

Figure S1. Kaplan–Meier curve for overall survival divided into two groups who had progression disease within three months and who did not.

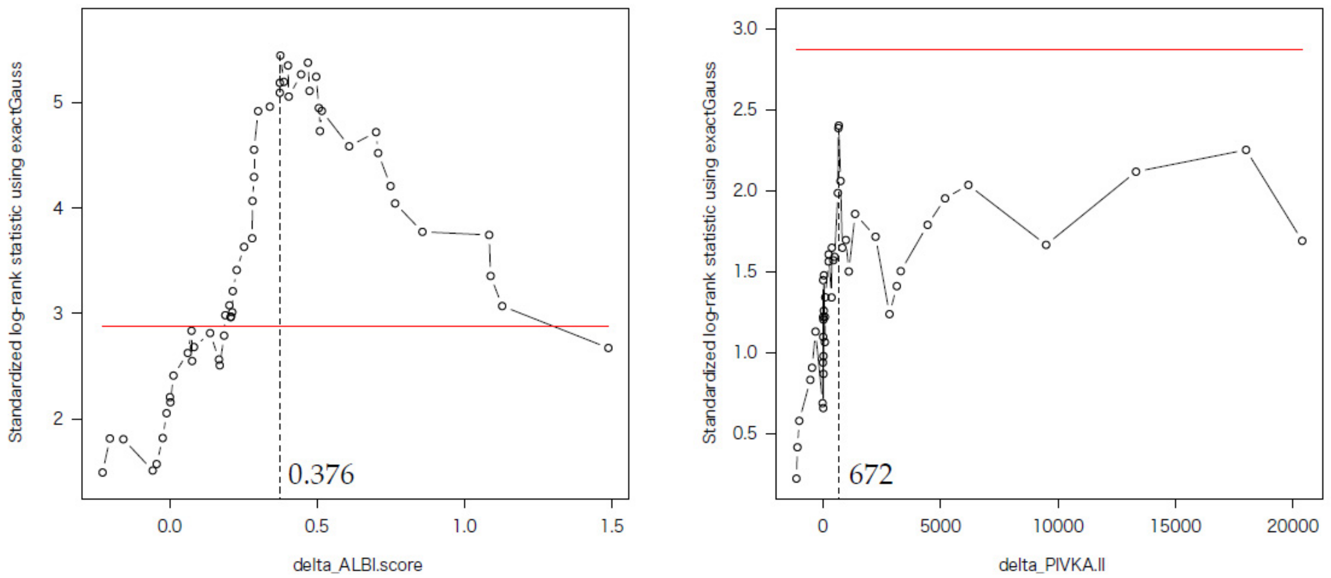

Figure S2. The result of the optimal cutoff values of the Δ ALBI score and Δ PIVKA-II according to the maximally selected rank statistics

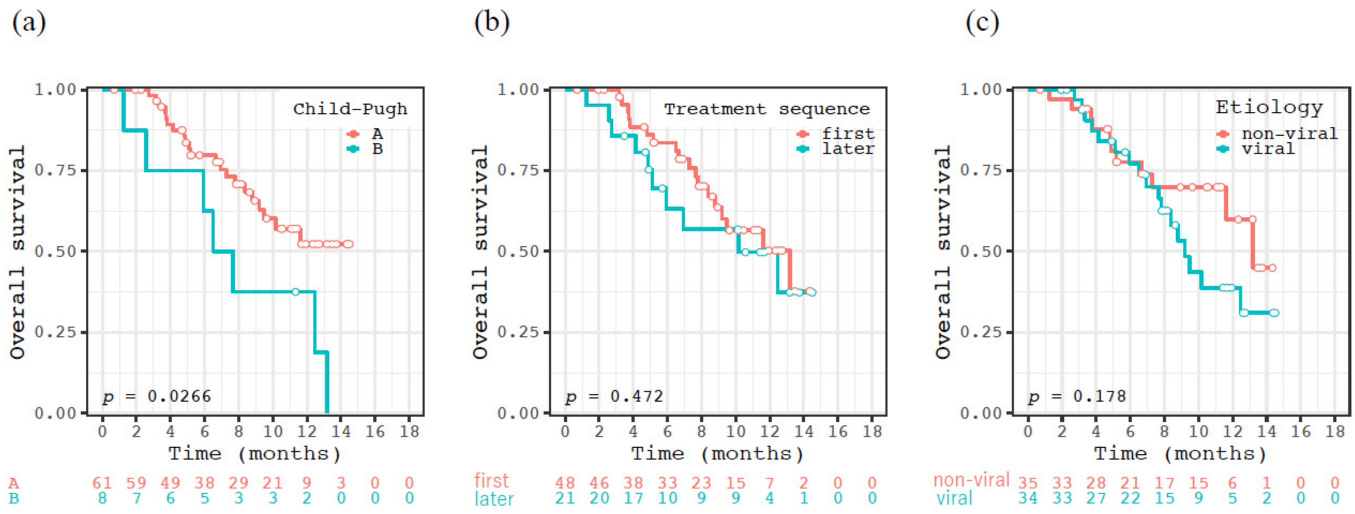

Figure S3: Kaplan–Meier curve for overall survival divided by Child–Pugh A and B (a), first-line and later-line (b), and viral hepatitis and non-viral hepatitis (c).

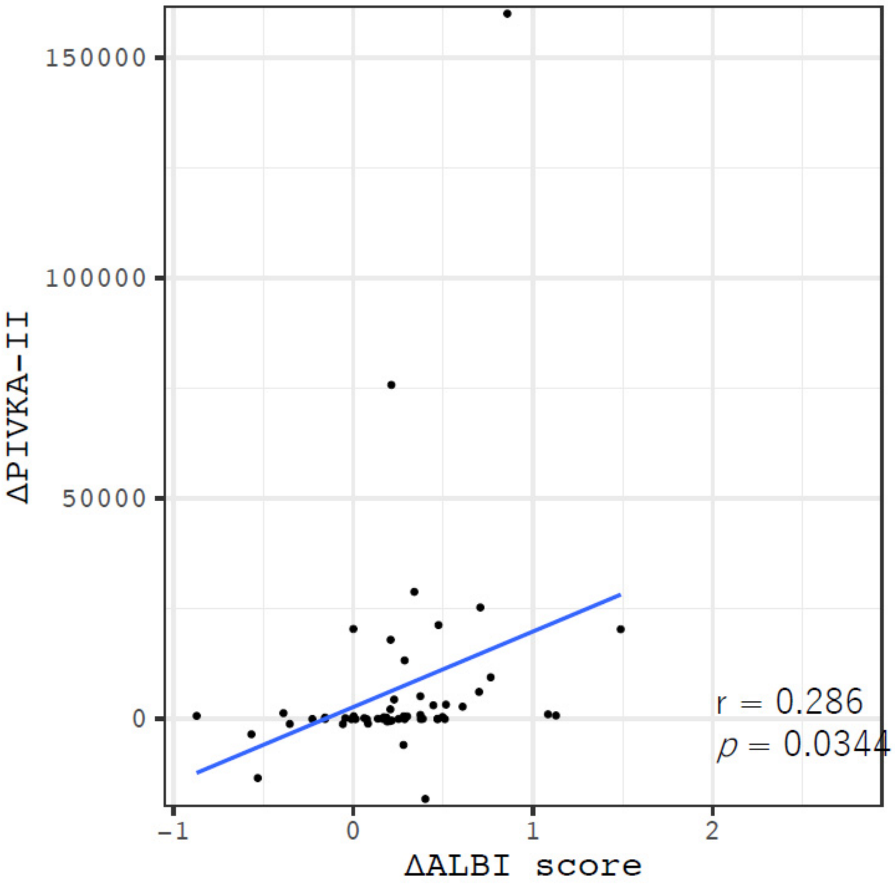

Figure S4. A correlation between the Δ ALBI score and Δ PIVKA-II.

Table S1. Univariate and mutivariate analyses of possible risk factors for overall survival among the changes of clinical indicators within 3 months by Cox proportional hazards model using AFP and PIVKA-II change ratio instead of Δ AFP and Δ PIVKA-II

Variables	univariate analysis		mutivariate analysis	
	**HR (95%CI)**	***P* value**	**HR (95%CI)**	***P* value**
Δ Child-Pugh score /3 months	1.971 (1.420–2.737)	<0.001		
Δ ALBI score /3 months	2.951 (1.956–4.453)	< 0.001	5.785 (1.822–18.37)	0.003
Δ Albumin (g/dL) /3 months	0.167 (0.069–0.409)	< 0.001		
Δ AST (U/L) /3 months	1.001 (0.991–1.011)	0.846		
Δ ALT (U/L) /3 months	0.999 (0.985–1.014)	0.925		
Δ T-Bil (mg/dL) /3 months	1.276 (1.092–1.490)	0.002		
Δ PT (%) /3 months)	0.985 (0.966–1.004)	0.120		
AFP change ratio*	1.025 (1.003–1.047)	0.023	1.023 (0.998–1.045)	0.110
PIVKA-II change ratio*	1.015 (0.996–1.033)	0.118		

* defined by AFP and PIVKA-II values at 3 months after Atez / Bev treatment divided by their values before the treatment

Table S2. Univariate and mutivariate analyses of possible risk factors for overall survival in 54 patients who did not have progression disease at 3 months

Variables	univariate analysis		mutivariate analysis	
	**HR (95%CI)**	***P* value**	**HR (95%CI)**	***P* value**
Δ Child-Pugh score /3 months	1.778 (1.236–2.557)	0.002		
Δ ALBI score /3 months	4.417 (2.171–8.985)	< 0.001	1.893 (0.422-8.491)	0.405
Δ Albumin (g/dL) /3 months	0.281(0.095–0.827)	0.021		
Δ AST (U/L) /3 months	1.030 (1.003–1.057)	0.027	1.028 (0.997-1.060)	0.075
Δ ALT (U/L) /3 months	1.037 (1.007–1.069)	0.016		
Δ T-Bil (mg/dL) /3 months	1.252 (1.072–1.461)	0.004		
Δ PT (%) /3 months)	0.982 (0.961–1.002)	0.080		
Δ AFP (ng/mL) /3 months	1.002 (0.999–1.004)	0.152		
Δ PIVKA-II (mAU/mL) /3 months	1.002 (1.001–1.003)	0.007	1.002 (1.000–1.003)	0.033
